# S-nitrosation of protein phosphatase 1 mediates alcohol-induced ciliary dysfunction

**DOI:** 10.1038/s41598-018-27924-x

**Published:** 2018-06-26

**Authors:** Michael E. Price, Adam J. Case, Jacqueline A. Pavlik, Jane M. DeVasure, Todd A. Wyatt, Matthew C. Zimmerman, Joseph H. Sisson

**Affiliations:** 1 From the Department of Internal Medicine, Pulmonary, Critical Care, Sleep & Allergy Division, University of Nebraska Medical Center, Omaha, NE USA; 2 Department of Cellular and Integrative Physiology, University of Nebraska Medical Center, Omaha, NE USA; 3 Department of Environmental, Agricultural, and Occupational Health, University of Nebraska Medical Center, Omaha, NE USA; 4 Nebraska-Western Iowa VA Healthcare System, Research Service, Omaha, NE USA

## Abstract

Alcohol use disorder (AUD) is a strong risk factor for development and mortality of pneumonia. Mucociliary clearance, a key innate defense against pneumonia, is perturbed by alcohol use. Specifically, ciliated airway cells lose the ability to increase ciliary beat frequency (CBF) to β-agonist stimulation after prolonged alcohol exposure. We previously found that alcohol activates protein phosphatase 1 (PP1) through a redox mechanism to cause ciliary dysfunction. Therefore, we hypothesized that PP1 activity is enhanced by alcohol exposure through an S-nitrosothiol-dependent mechanism resulting in desensitization of CBF stimulation. Bronchoalveolar S-nitrosothiol (SNO) content and tracheal PP1 activity was increased in wild-type (WT) mice drinking alcohol for 6-weeks compared to control mice. In contrast, alcohol drinking did not increase SNO content or PP1 activity in nitric oxide synthase 3-deficient mice. S-nitrosoglutathione induced PP1-dependent CBF desensitization in mouse tracheal rings, cultured cells and isolated cilia. *In vitro* expression of mutant PP1 (cysteine 155 to alanine) in primary human airway epithelial cells prevented CBF desensitization after prolonged alcohol exposure compared to cells expressing WT PP1. Thus, redox modulation in the airways by alcohol is an important ciliary regulatory mechanism. Pharmacologic strategies to reduce S-nitrosation may enhance mucociliary clearance and reduce pneumonia prevalence, mortality and morbidity with AUD.

## Introduction

Alcohol abuse is associated with 80,000 deaths and a ~$240 billion economic loss per year in the United States^1,2^. Contributing to this mortal and economic burden is the long-established association of alcohol use disorder (AUD) with increased prevalence and severity of pneumonia^3^. Individuals with AUD are approximately 3 times more likely to develop pneumonia. Moreover, patients with AUD that develop pneumonia are twice as likely to develop sepsis, and are also at increased risk to develop acute respiratory distress syndrome^4^. One of the first lines of defense of the lung and airways against pneumonia causing pathogens is mucociliary clearance^5^. Key to mucociliary clearance is the coordinated movement, or beating, of motile cilia functioning as an escalator to propel mucus-trapped pathogens out of the airway. These cilia maintain a baseline resting frequency that, upon stimulation by mechanical or chemical stimuli, rapidly increases to improve clearance^6^. Our recent studies showed that prolonged alcohol exposure results in dysfunction of the signaling pathway that activates this increase in ciliary beat frequency (CBF) possibly driven by the redox post-translational modification S-nitrosation of protein phosphatase 1 (PP1)^7–9^.

Regulation of CBF by alcohol exposure occurs in two phases; a rapid and transient stimulation of CBF followed by desensitization of CBF stimulation to alternative stimuli. Initial stimulation of CBF by alcohol requires activation of sequential nitric oxide (^·^NO) and cAMP-dependent pathways that are intrinsic to cilia organelles, and occurs independent of the cell^10^. Upon initial exposure, alcohol increases CBF by a ^·^NO-dependent mechanism requiring the endothelial isoform of ^·^NO synthase (eNOS/NOS3) and heat shock protein 90 (HSP90)^11^. Increased ^·^NO production activates cilia-localized soluble guanylyl cyclase (sGC) driving formation of 3′, 5′-cyclic guanosine monophosphate (cGMP). Simultaneously, by an unknown mechanism, alcohol increases soluble adenylyl cyclase (sAC) formation of 3′, 5′-cyclic adenosine monophosphate (cAMP)^12^. Sequential activation of cGMP-dependent protein kinase (PKG) and then cAMP-dependent protein kinase (PKA) results in increased phosphorylation of an unknown 29-kDa protein and increased CBF^8,10^. PKA activation is a final common mediator for several signal transduction pathways that result in increased CBF, including adrenergic agonists^6,10,13^. After a transient increase following brief exposure to alcohol, CBF returns to baseline despite the continued presence or addition of alcohol^14,15^.

In contrast, sustained alcohol exposure activates protein phosphatase 1 (PP1) preventing PKA activation and stimulation of CBF by β-adrenergic agonists^8,9^, which we have termed alcohol-induced ciliary dysfunction (AICD). Specifically, we recently identified that PP1 cysteine 155 (PP1^C155^) is oxidized in bovine airway axonemes after alcohol exposure, which correlated with the desensitization of isolated axoneme CBF to cAMP. Importantly, oxidation of PP1, and CBF desensitization can be reversed by ascorbate, an antioxidant relatively selective for S-nitrosation^16^. The objective of the current study is to investigate the potential for alcohol to drive S-nitrosation *in vivo* and precisely define the role of PP1 to regulate stimulated CBF under oxidizing conditions such as alcohol exposure. We hypothesized that alcohol exposure drives an increase in PP1 activity by S-nitrosation of PP1^C155^ resulting in desensitization of CBF. We report that alcohol drinking increases bronchoalveolar S-nitrosothiol (SNO) content and PP1 activity in WT mice that is not apparent in NOS3^−/−^ mice. Furthermore, our results suggest that direct S-nitrosation of PP1 increases enzymatic activity and that oxidation of cysteine 155 is a key component in the alcohol-driven desensitization of CBF stimulation.

## Results

### S-nitrosoglutathione drives protein phosphatase 1-dependent cilia dysfunction in bovine axonemes

**Figure 1:**
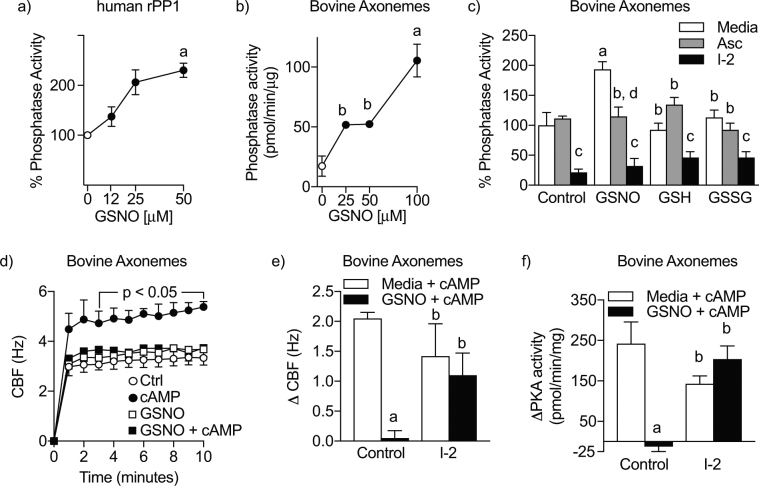
GSNO drives PP1 activity to block cAMP-responsiveness in bovine axonemes. (**a**) S-nitrosoglutathione (GSNO) dose-dependently (0–100 μM; 10 minutes) increases recombinant human PP1 activity. ^a^p < 0.05 compared to 0 μM GSNO, n = 3. (**b**) GSNO (0–100 μM; 10 minutes) dose-dependently increases protein phosphatase activity in isolated bovine axonemes. ^a^p < 0.01 compared to 0 μM GSNO, ^b^p < 0.05 compared to 100 uM GSNO. n = 3 axoneme preparations. (**c**) GSNO activation of PP1 in bovine axonemes is nitrosothiol-dependent. ^a^p < 0.001 compared to media control, ^b^p < 0.001 compared to media GSNO, ^c^p < 0.001 compared to Asc of same condition. n = 3–4 axoneme preparations. (**d**,**e**) GSNO (100 μM; at time of reactivation) blocks cAMP-dependent CBF responsiveness, which is reversed by I-2. ^a^p < 0.0001 compared to media control; ^b^p < 0.005 compared to control GSNO. n = 5 axoneme preparations. (**f**) I-2 (2.0 nM; 5 minutes pre-GSNO) restores cAMP-dependent PKA-responsiveness to GSNO treated axonemes. ^a^p < 0.0001 compared to media control. ^b^p < 0.01 compared to control GSNO. n = 5 axoneme preparations. rPP1 = recombinant protein phosphatase 1, GSH = reduced glutathione, GSSG = oxidized glutathione, Asc = ascorbate, I-2 = inhibitor 2.

We hypothesized that SNO donors activate PP1. To determine if PP1 activity was directly modified by redox state, we measured the activity of purified recombinant human PP1 or PP1 activity in isolated bovine axonemes after direct 10-minute incubation with the SNO donor, S-nitrosoglutathione (GSNO). PP1 activity was enhanced up to two-fold in a dose responsive manner to micromolar concentrations of GSNO both in purified PP1 and bovine axonemes (Fig. 1a,b). We next sought to determine the redox specificity of PP1 activation by GSNO. Incubation with ascorbate (Asc), an antioxidant that shows specificity for reduction of S-nitrosothiols, returned GSNO-driven bovine axoneme PP1 activity back to baseline. Importantly, reduced glutathione (GSH) and oxidized glutathione (GSSG) equivalents did not result in PP1 activation, indicating the necessity of a nitrogen oxide in GSNO-mediated PP1 activation. Incubation with inhibitor-2 (I-2), a highly selective recombinant PP1 inhibitor, completely inhibited phosphatase activity (Fig. 1c). These data indicate that PP1 activity is enhanced by S-nitrosation upon incubation with GSNO.

To identify the role S-nitrosation plays in regulating ciliary motility, we measured CBF in a bovine ATP-reactivated isolated ciliary motility system. We hypothesized that GSNO activation of PP1 inhibits cAMP-dependent stimulation of CBF. To test this hypothesis, we incubated bovine axonemes with ± GSNO (at time of reactivation) and ± I-2 (5 minute pre-incubation). GSNO (100 μM) alone did not change baseline CBF (Fig. 1d). After assessing baseline CBF, we then determined ciliary responsiveness by adding cAMP at time of reactivation to activate PKA. Cyclic AMP-dependent increases in CBF and PKA activity were blocked by pre-incubation of bovine axonemes with 100 μM GSNO (Fig. 1d–f). Importantly, both CBF and PKA activity were restored by incubation with the PP1 inhibitor, I-2. These data indicate that GSNO-induced CBF desensitization is specifically driven by activation of PP1.

### Alcohol drinking drives bronchoalveolar S-nitrosothiols and tracheal ring PP1 activity in mice

**Figure 2:**
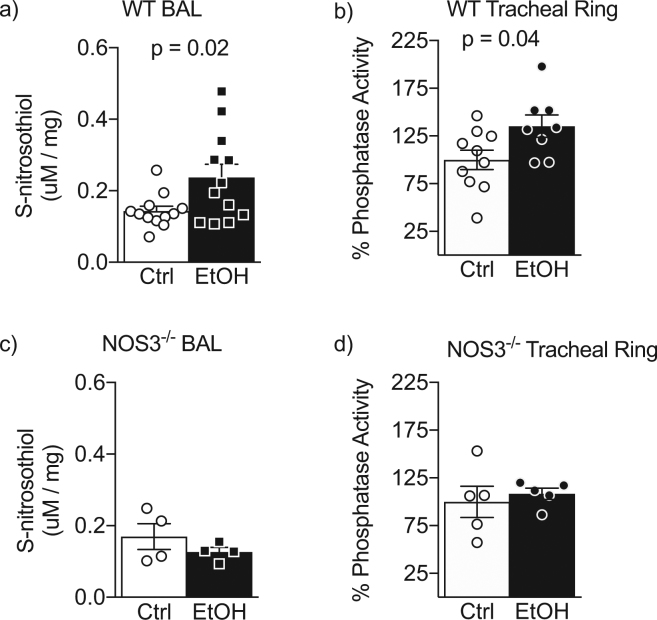
Alcohol drinking drives NOS3-dependent airway S-nitrosothiol production and PP1 activity in mice. (**a**,**b**) Alcohol drinking (6 weeks × 20% in drinking water) increases bronchoalveolar lavage (BAL) s-nitrosothiol (SNO) (**a**) and tracheal ring PP1 activity (**b**) in WT mice. n = 8–12 per group. (**c**,**d**) Alcohol does not increase BAL SNO (**c**) nor PP1 activity (**d**) in NOS3^−/−^ mice. n = 4 per group BAL SNO and n = 5 per group PP1 activity. P-values are from Student’s t-test; bars represent mean ± SEM.

We next sought to determine how alcohol drinking influenced SNO content and PP1 activity in an animal drinking model. We hypothesized alcohol drinking would increase both SNO content and PP1 activity after alcohol drinking in a NOS3-dependent manner. To test this hypothesis, we fed WT and NOS3^−/−^ C57BL/6 mice alcohol (20% by volume in drinking water) for 6 weeks and measured SNO content in bronchoalveolar lavage and PP1 activity in tracheal rings. Alcohol drinking increased bronchoalveolar lavage (BAL) SNO content and PP1 activity in WT alcohol drinking mice compared to WT mice that did not receive alcohol in their water. Importantly, tracheal ring PP1 activities and BAL SNO contents were not different in alcohol-drinking NOS3^−/−^ mice compared to NOS3^−/−^ mice that did not drink alcohol (Fig. 2a,b) indicating that NOS3 plays an important role in the activation of PP1 by alcohol exposure.

### S-nitrosoglutathione recapitulates AICD in mouse airway epithelium

**Figure 3:**
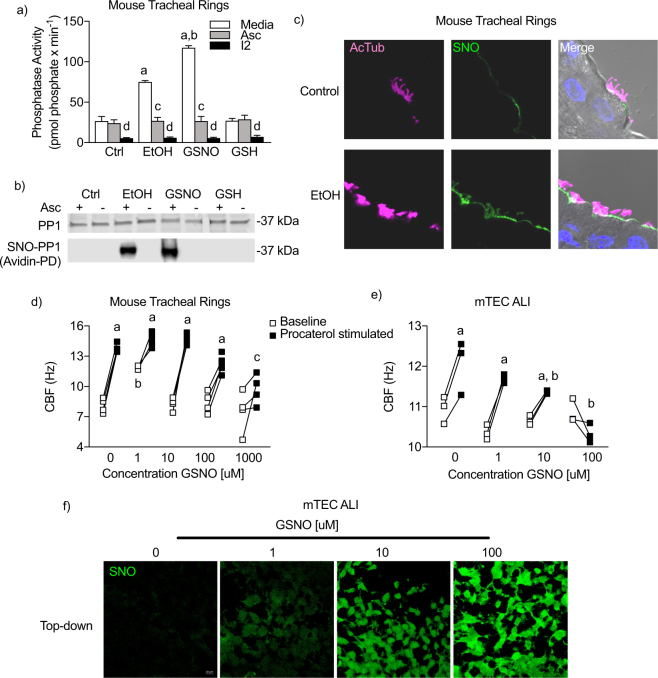
GSNO recapitulates AICD in mouse tracheal rings. (**a)** GSNO 4 h × 1 mM) and alcohol (EtOH; 100 mM × 10 days) increase PP1 activity in mouse tracheal rings, which is reversed by ascorbate (10 min × 30 μM). Data are representative of the mean of 3–6 tracheal rings from 4 mice. ^a^p < 0.05 compared to media control. ^b^p < 0.005 compared to EtOH. ^c^p < 0.05 compared to media within group. ^d^p < 0.05 compared to Asc within group. (**b**) GSNO and EtOH increase biotin switch detection of PP1 in mouse tracheal rings. The absence of Asc is a negative control for the biotin switch assay. (**c**) *In vitro* EtOH increases cilia S-nitrosation in mouse tracheal rings. Acetylated tubulin (AcTub) - pink; Avidin-fluorescein (SNO) - green (**d**) GSNO (4 h) blocks procaterol (1 h × 10 nM) stimulation in mouse tracheal rings. ^a^p < 0.0001 compared to baseline of the same condition. ^b^p < 0.001 compared to control baseline. ^c^p < 0.01 compared to control stimulated. Data are representative of 3–4 tracheal rings from 4–5 mice per condition. (**e**) GSNO (4 h) blocks procaterol stimulation in cultured mouse tracheal epithelial cells. ^a^p < 0.001 compared to baseline of the same condition. ^b^p < 0.001 compared to control stimulated. n = 13–24 cultures per group. (**f**) GSNO (4 h) increases S-nitrosation in mouse tracheal epithelial cells. *Top*: Looking down on ALI culture. Avidin-fluorescein (SNO) – green.

Our next objective was to determine the role of alcohol-driven SNO on PP1 activity and CBF activity in samples from mice *in vitro*. We hypothesized that GSNO and alcohol produce ciliary dysfunction in mouse tracheal airway epithelial cells. To test this hypothesis, we treated tracheal rings from naïve WT mice for 10 days with 100 mM alcohol or 4 hours with 100 μM GSNO and compared phosphatase activity and S-nitrosation to control. Alcohol and GSNO exposure resulted in increased PP1 activity and S-nitrosation in mouse tracheal rings (Fig. 3a,b). *Ex vivo* treatment of tracheal ring lysates with ascorbate returned PP1 activity to baseline in alcohol- or GSNO-exposed tracheal rings (Fig. 3a). Importantly, GSH did not stimulate phosphatase activity and minimal phosphatase activity was detected when lysates were treated with I-2 indicating specificity for nitrogen oxide and PP1, respectively (Fig. 3a). Moreover, GSNO and alcohol both increased S-nitrosation of PP1 as detected by the biotin switch assay (Fig. 3b). In alcohol-treated tracheal rings, SNO-labeling (avidin-fluorescein, SNO; green) colocalized with cilia (acetylated tubulin, AcTub; pink). However, only a small of amount of SNO detection was present on the apical surface of airway epithelial cells that did not colocalize with cilia in control tracheal rings (Fig. 3c). We next assessed the effects of GSNO on CBF. Baseline CBF was changed by GSNO and CBF responsiveness dose-dependently decreased with increasing concentrations of GSNO (Fig. 3d). Interestingly, a low dose of GSNO (1 μM) stimulated CBF from baseline (Fig. 3d) compared to control-treated tracheal rings, consistent with previously reported data^17^. This stimulatory response did not persist at higher concentrations of GSNO (10–1000 μM). We next assessed the role of GSNO in a cell culture model of mouse tracheal epithelial cells (mTECs) cultured at air-liquid interface. GSNO exposure had no effect on baseline CBF in mTECs at 4 hours. However, GSNO exposed mTECs showed a dose-responsive decrease in responsiveness to 10 nm of the β-agonist procaterol, with complete loss of responsiveness at 100 μM GSNO (Fig. 3e). Additionally, GSNO dose dependently increased cilia-localized S-nitrosation (Fig. 3f). In combination, these data demonstrate that cilia and cilia-localized PP1 are sensitive to S-nitrosation and that alcohol can drive S-nitrosation in ciliated airway epithelial cells.

### Human airway epithelial cell cilia motility is desensitized to β-agonist driven CBF responsiveness by alcohol exposure

**Figure 4:**
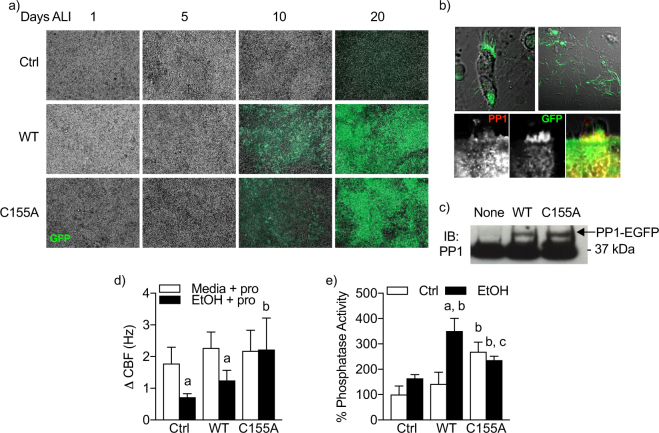
Mutagenesis of PP1 cysteine 155 protects against AICD in cells. (**a**) Foxj1-driven PP1 expression is consistent with differentiation of human airway epithelial cells. Green = GFP. 10X magnification. (**b**) *Left*, Foxj1-driven PP1eGFP expression localizes to the nucleus and cilia in an intact cell from a hBEC ALI culture. *Right*, PP1eGFP fluorescence persists in isolated demembranated axonemes from an hBEC ALI culture. *Bottom*, PP1 and GFP colocalize. (**c**) Western blot for PP1α in control, PP1^WT^-EGFP and PP1^C155A^-EGFP. D) Change in CBF (Δ CBF) pre and post-1 hr procaterol (pro) treated ± alcohol (24 h × 100 mM). ^a^p < 0.05 compared to no alcohol within same PP1 genotype. ^b^p < 0.05 compared to media ctrl. Data are representative of mean CBF of 3 cultures with at least 6 CBF readings per condition for at least 3 experiments. (**e**) Phosphatase activity from whole cell lysates of human airway epithelial cells treated as in D. ^a^p < 0.05 compared ctrl of same genotype. ^b^p < 0.05 compared to no alcohol non-transduced control. ^c^p < 0.05 compared to WT EtOH.

We previously reported in a bovine isolated cilia model that alcohol drives S-nitrosation of PP1 at cysteine 155 (PP1^C155^)^9^. Moreover, PP1 contains a putative redox regulatory domain that consists of cysteine 155 and cysteine 158^18^. The sequence of PP1 is 99% conserved between humans, mice and cattle. We hypothesized that mutagenesis of human PP1^C155^ to alanine (PP1^C155A^) would prevent PP1 activation by alcohol exposure in human airway epithelial cells. To test this hypothesis, we generated HIV1 lentiviral constructs with the ciliated cell-specific transcription factor *foxj1* harboring sequences for human wild-type-PP1 (PP1^WT^) or PP1^C155A^ fused with enhanced green fluorescence protein (EGFP) and overexpressed these proteins in human ciliated airway epithelial cells cultured at air-liquid interface. EGFP fluorescence was coincident with the appearance of motile cilia between days 14–20 of air-liquid interface (Fig. 4a). PP1 and EGFP expression localized primarily to the nucleus and to cilia, co-localized with staining for PP1 (Fig. 4b, Movie S1) and appeared at the predicted molecular weight (~70 kDa) by electrophoresis (Fig. 4c). The transduction efficiency was nearly 100% in ciliated cells. After establishing these cultures and confirming PP1 expression, we then treated control, PP1^WT^ and PP1^C155A^ transduced cells with 100 mM alcohol for 24 hours followed by treatment with procaterol for 1 hour. Baseline CBF was not different between the groups after 24 hours of alcohol exposure (Fig. 4d). Control and PP1^WT^ alcohol-treated cells had a diminished response to the β-agonist procaterol compared to cells receiving media alone (Fig. 4d). However, cultures transduced with PP1^C155A^ demonstrated an increased CBF after 1 h procaterol compared to control and PP1^WT^ cultures exposed to alcohol (Fig. 4d). Phosphatase activity was only slightly elevated in non-transduced cultures after alcohol exposure compared to control (Fig. 4e). However, in PP1^WT^ cultures, alcohol stimulated phosphatase activity approximately 2-fold compared to control of the same PP1^WT^ culture. Baseline phosphatase activity in PP1^C155A^ cultures was higher than non-transduced cells without alcohol, but alcohol did not further increase phosphatase activity in PP1^C155A^ cultures (Fig. 4e).

### Alcohol-driven PP1 activation persists in isolated axonemes from cultured human airway epithelial cells

**Figure 5:**
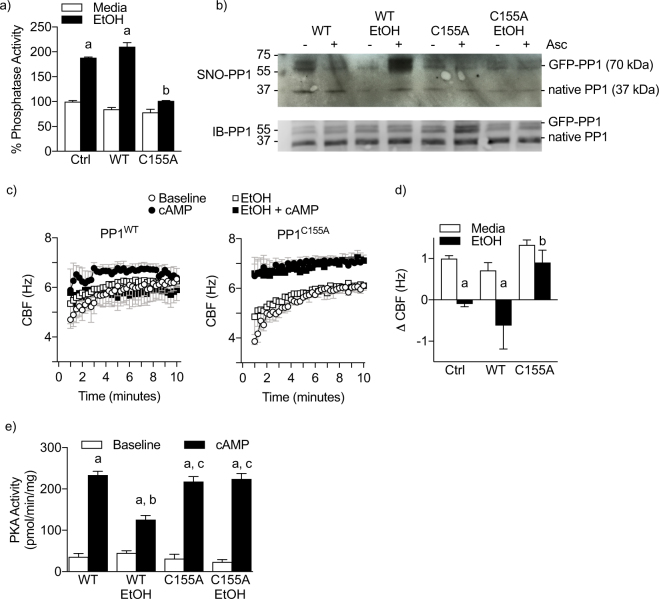
Mutagenesis of PP1 cysteine 155 reverses AICD in isolated axonemes from human airway epithelial cells. (**a**) Alcohol (24 h × 100 mM) does not increase phosphatase activity in axonemes expressing C155A mutant PP1. ^a^p < 0.05 compared to media of same genotype; ^b^p < 0.05 compared to Ctrl EtOH or WT EtOH; n = 6−7. (**b**) C155A mutagenesis prevents the majority of S-nitrosation of axonemal PP1. The absence of Asc is a negative control for the biotin switch assay. (**c**) Representative motility of isolated axoneme motility for 10 minutes following addition of ATP. *Left*, Axonemes isolated from hBECs expressing PP1^WT^. *Right*, Axonemes isolated from hBECs expressing PP1^C155A^ (**d**) C155A mutagenesis restores cAMP-dependent stimulation of CBF in isolated axonemes from hBECs treated with alcohol. ^a^p < 0.05 compared to media ctrl; ^b^p < 0.05 compared to Ctrl EtOH or WT EtOH. n = 3. (**e**) PP1^C155A^ restores PKA responsiveness in isolated axonemes from hBECs treated with alcohol. ^a^p < 0.05 compared to baseline of same genotype; ^b^p < 0.05 compared to EtOH Ctrl or WT, ^c^p < 0.05 compared WT EtOH; n = 4.

Since immunofluorescence revealed PP1^WT^ and PP1^C155A^ expression to be distributed in multiple locations throughout the cell, we sought to determine if S-nitrosated PP1 regulated cilia motility at the level of the axoneme or was based on an intact cell-dependent process. We hypothesized that axonemes isolated from human airway epithelial cells treated with alcohol would retain a desensitized phenotype (blunted PKA and CBF responses to cAMP). To test this hypothesis, we measured phosphatase activity in isolated axonemes from cells transduced with or without PP1^WT^ or PP1^C155A^ and treated with or without alcohol (100 mM × 24 h). Alcohol treatment stimulated phosphatase activity and PP1 S-nitrosation in PP1^WT^ cultures, but not in PP1^C155A^ expressing cultures (Fig. 5a,b). Additionally alcohol stimulated S-nitrosation of PP1^WT^ cultures compared to the no alcohol controls (Fig. 5b). When the biotin switch technique for s-nitrosation was performed on these samples in the absence of ascorbate, a reagent relatively specific for cleavage of the SNO bond necessary to generate free thiols from SNO bonds to be biotinylated, there was little to no detection of biotin compared to when ascorbate was present. This suggests the biotin signal in PP1^WT^ cultures exposed to alcohol is specific for S-nitrosation. Importantly, expression of PP1^C155A^ completely abrogated detection of alcohol-driven S-nitrosation by the biotin switch technique (Fig. 5b). These data suggest that the primary site of S-nitrosation of PP1 is C155.

All of the necessary machinery for ciliary bending is localized to the axoneme^10^. Upon isolation, axonemes can be reactivated with exogenous ATP to reinitiate beating. Additionally, PKA is localized to the axoneme and exogenous cAMP stimulates isolated axoneme motility^10,13^. We previously established that the phenomenon of AICD (PKA and CBF desensitization) persists at the level of the isolated axoneme in bovine axonemes^9^. Since PP1 is present in isolated axonemes (Fig. 4b, Supplementary Movie S2), we next sought to understand if mutagenesis of PP1 was sufficient to restore PKA and CBF responsiveness in human isolated axonemes. We hypothesized that PP1^C155A^ prevents PP1 activation and, therefore, CBF desensitization by alcohol. To test this hypothesis, we performed isolated axoneme motility experiments from axonemes isolated from PP1^WT^- and PP1^C155A^-expressing human bronchial epithelial cell (hBEC) cultures treated with or without alcohol (100 mM × 24 h). Isolated axonemes from untreated PP1^WT^ and PP1^C155A^ cultures maintained a similar baseline CBF after reactivation with ATP (Fig. 5c). We next stimulated axonemes from control or alcohol treated PP1^WT^ or PP1^C155A^ cultures with cAMP. Cyclic AMP (at time of reactivation) stimulated CBF comparably in PP1^WT^ and PP1^C155A^ cultures that did not receive alcohol treatment (Fig. 5c). This cAMP stimulation was abolished in axonemes extracted from PP1^WT^-expressing cultures that were treated with alcohol. Importantly, axonemes from PP1^C155A^ alcohol-treated cells retained responsiveness to cAMP (Fig. 5c). The difference between cAMP-stimulated and baseline CBF (ΔCBF) after 10 minutes for each condition are demonstrated in Fig. 5d. Additionally, we measured PKA activity directly. In PP1^WT^ axonemes extracted from alcohol-treated cells, PKA responsiveness to cAMP was blunted compared to axonemes extracted from control-treated cells (Fig. 5e). PP1^C155A^ mutagenesis prevented this PKA desensitization (Fig. 5e).

## Discussion

It is well established that alcohol abuse is an independent risk factor for the development of costly and difficult to manage pneumonias^19^. Indirect evidence, such as the depletion of key antioxidants, indicates the generation and imbalance of reactive oxygen species in alcohol-associated lung injury^20–22^. Here we have identified that alcohol drinking in a mouse model increases SNO content in the airways. Moreover, using several approaches, we identified PP1 as a downstream effector of increased S-nitrosation driven by alcohol. These are the first data to produce AICD *in vitro* in human airway epithelial cells, demonstrating the potential clinical importance of alcohol toward cilia dysfunction in humans. Additionally, these are the first data describing a novel human isolated axoneme motility system to manipulate PP1 and measure CBF. To our knowledge, these are the first data reporting baseline and cAMP-stimulated CBF from human isolated axonemes. Using this isolated human axoneme system; our data demonstrate that a highly conserved cysteine (cysteine 155) mediates oxidant-induced activation of PP1 in human airway epithelial cells exposed to alcohol. These data corroborate previous *in silico* studies identifying cysteine 155 and 158 as existing in a thioredoxin-like tertiary structure^18^. Additionally, these data provide definitive evidence that PP1 localizes to the axoneme in human airway epithelial cells and is a key factor in AICD.

The metabolism of ethanol drives the consumption of NAD^+^, an important cofactor for maintaining a reducing environment within the cell^22^. Additionally, high doses of ethanol increase ^·^NO and production of ethyl nitrite, a potent S-nitrosating agent^23^. Indeed, we have previously identified that alcohol exposure drives increased SNO content of bovine airway axonemes and alcohol-driven S-nitrosation correlates with activation of PP1^9^. These data are further corroborated by our finding that alcohol-drinking results in increased BAL SNO content compared to controls. Importantly, this increase is not apparent in NOS3^−/−^ mice drinking alcohol (Fig. 2c), implicating the clear role of ^·^NO production in the nitrosation-dependent pathway. In addition to these data, previous data demonstrated that concomitant feeding of antioxidants and alcohol prevents AICD^7^. These data in combination suggest a nitroso-redox mechanism for AICD mediated by PP1.

In addition to our work, Sommer *et al*. demonstrated a substantial increase in purified PP1 activity after incubation with a xanthine oxidase oxidant-generating system^24^. This effect seemed to be mediated by H_2_O_2_ as catalase prevented PP1 activation and superoxide dismutase did not^24^, indicating PP1 activation by oxidation. Our data suggest that the PP1 catalytic subunit can be directly activated by GSNO (Fig. 1a). In line with this, the tertiary structure of PP1 contains a putative redox active site consisting of cysteine 155 and 158^18^. When we expressed a PP1^C155A^ mutant, alcohol no longer stimulated PP1 activity or resulted in desensitization of β-agonist stimulated CBF (Fig. 4d) at the cellular and isolated axoneme levels (Fig. 5c). Interestingly, overexpression of PP1 alone was not sufficient to cause cilia desensitization. This suggests that the activation of PP1 by alcohol-exposure is driven by a post-translational modification, or alters disulfide bridging with a regulatory protein.

Our study is limited by the use of animal models and *in vitro* cell culture systems. If and how alcohol consumption drives SNO production in living humans has yet to be determined. Another limitation to extending our findings to a treatment strategy is that PP1 is a ubiquitous enzyme necessary for many cellular functions beyond regulation of CBF including apoptosis and cell division^25^. Thus, direct targeting of PP1 is likely an inefficient strategy to rescue AICD clinically. Additionally, targeting SNOs may prove equally challenging due to off-target effects.

Our finding that alcohol drives SNOs may have implication beyond CBF and mucociliary clearance. Mice that are deficient in the primary enzyme to metabolize SNOs, S-nitrosoglutathione reductase (GSNOR), demonstrate increases in two diseases clinically correlated with alcohol abuse; Klebsiella pneumonia and hepatocellular carcinoma (HCC). In a mouse model of pneumonia, after inoculating with *Klebsiella pneumoniae*, Tang *et al*. observed a 4-fold increase in lung bacterial burden, an over 1000-fold increase in blood burden and had a 40% mortality rate compared to wild-type mice that had a 0% mortality rate^26^. Data reported by Wei *et al*. demonstrated that GSNOR^−/−^ mice developed spontaneous HCC 10 times more frequently than wild-type controls^27^. In addition, in a small study of 24 patients with HCC, GSNOR was found to be about 50% decreased in cancerous liver tissue compared to noncancerous tissue from the same donor^27^. In the context of our data, S-nitrosation is implicated as an important target in the study of alcohol tissue injury.

Clinical trials aimed at increasing airway S-nitrosothiols, by inhibiting S-nitrosoglutathione reductase (GSNOR), are underway to enhance surface expression of cystic fibrosis transmembrane conductance regulator as treatment for cystic fibrosis. *In vitro* data suggest that exogenous GSNO enhances Cl^−^ currents, an *in vitro* readout that generally correlates well to a clinical improvement^28^. Despite these exciting findings with GSNOR inhibitors, clinical trials for these drugs have failed to meet efficacy. It is possible that GSNOR inhibitors promote PP1 activation and cilia desensitization. Future studies should examine the role of GSNOR inhibitors on CBF.

**Figure 6:**
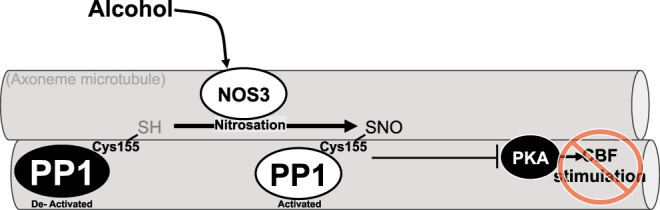
Regulation of PP1 and CBF stimulation by alcohol and S-nitrosation. Alcohol activates Nitric Oxide Synthase 3 to promote S-nitrosation of Protein Phosphatase 1 (PP1) at cysteine 155 (Cys155). S-nitrosation of PP1 at Cys155 activates PP1 preventing activation of cilia-localized cyclic AMP-dependent protein kinase (PKA) and stimulation of ciliary beat frequency (CBF).

In summary, we have demonstrated that alcohol drinking drives airway S-nitrosation. Cysteine 155 is critical for the activation of PP1 in the context of SNO donors and prolonged alcohol exposure, which occurs in bovine isolated axonemes, mouse tracheal tissue and cultured cells, cultured primary human airway epithelial cells and isolated human axonemes. Activation by S-nitrosation of PP1 prevents PKA activation and stimulation of CBF (Fig. 6). Importantly, these data highlight S-nitrosation as a key component of PP1 activation and cilia desensitization at the organelle level in human samples.

## Methods

### Mouse model of alcohol drinking

All experiments were reviewed and approved by the Institutional Animal Care and Use Committee (IACUC) of the University of Nebraska Medical Center. All methods were performed in accordance with the relevant guideline and regulations. Female C57BL/6 mice, purchased from Charles Rive Laboratories (Wilmington, MA) were acclimated to the animal facility at the University of Nebraska Medical Center and given water or 20% ethyl alcohol to drink as previously described^7^. In brief, mice were given increasing concentrations of ethanol in water over a 1-week period until the target concentration of 20% was reached. Mice in the alcohol group were given 5% alcohol (w/v) to drink *ad libitum* (95% ethanol diluted with Milli-Q water) for 2 days, 10% ethanol (w/v) for 2 days, 15% ethanol (w/v) for 3 days, and 20% ethanol (w/v) for 6 weeks. Saccharin (0.1–0.5% w/v) was added to the water in all groups. Mice in the matched control group were given water from the same source without ethanol. Additionally, female NOS3^−/−^ and WT littermate control C57BL/6 mice (Jackson Laboratories, Bar Harbor, ME) were given alcohol or water to drink as described above. We chose female mice based on previously published reports that female mice express more lung NOS3 than their male counterparts^29^ and based on previous reports of AICD^7^.

### Determination of S-nitrosothiols in bronchoalveolar lavage fluid

At the end of the treatment period, each mouse was sequentially sacrificed by isoflurane (Medline Industries; Northfield, IL) followed immediately by whole lung lavage with 1.0 mL sterile normal saline. Mice and fluids were handled out of direct sunlight in a low-lit room. 100 μl of the fluid was centrifuged at 1000 × g for 5 minutes and the supernatant added to 900 μl 10X solution of 5.0 mM EDTA, 1.0 mM DPTA, 0.1 mM neocuproine in a black tube, immediately frozen in liquid nitrogen and stored at −80 °C for up to 3 months. SNO content was determined by combination of the Griess assay with and without incubation of the sample with mercury chloride (HgCl_2_) as previously described using a modification of the Cayman Nitrate/Nitrite Colorimetric Assay Kit (Cayman Chemical; Ann Arbor, MI)^9^. SNO content was determined by subtracting the amount of nitrite in a sample incubated in the absence of HgCl_2_ from the same sample incubated in the presence of HgCl_2_ and normalized to the protein content of the sample determined by the Bradford Assay. Samples with proteins below the range of detection were excluded from the analysis.

### Isolation of bovine axonemes

No live animals were used for the collection of bovine axonemes. Excess tissue from a local abattoir was received under agreement and approval from the U.S. Department of Agriculture for use in this study. Axonemes were isolated from ciliated epithelium of bovine tracheae as previously described^10^.

### *Ex vivo* mouse tracheal ring culture

Tracheal rings from C57BL/6 mice obtained from Jackson Labs were cut and cultured as previously described^8^. At least 12 tracheal rings were cut from each mouse and each ring was randomized to a treatment group within the same experiment. The tracheal rings were treated with 100 mM alcohol for 10 days as previously described^8^. For GSNO experiments, tracheal rings were cultured in media alone for 9 days and 20 h followed by 4 h treatment with or without GSNO concentrations as indicated in Fig. 4c. For procaterol (Sigma, St. Louis, MO) treatment, a concentrated stock was added to the incubation medium for a final concentration 10 nM.

### Airway epithelial cultures at air-liquid interface

Tracheal epithelial cells from C57BL/6 mice obtained from Jackson Labs were collected and cultured at air-liquid interface as previously described^8^. After 14–21 days at ALI, a baseline CBF reading was taken and then cells were immediately treated with varying concentrations of S-nitrosoglutathione (0, 1, 10, or 100 μM; Enzo Life Sciences, Farmingdale, NY) by diluting a concentrated stock of GSNO to the basal medium and then adding 25 μl basal medium to the apical surface and placing back in the incubator. Primary, normal human bronchial epithelial cells (NHBEs) were isolated from de-identified human lungs that were not suitable for transplantation. We accepted lungs from the International Institute for the Advancement of Medicine (IIAM) and the Nebraska Organ Retrieval System (NORS). We excluded donors with a history of any lung disease, current smoking, ≥20 pack-year history of smoking, and heavy alcohol use. The protocol was approved by IIAM and NORS ethics committees and the University of Nebraska Medical Center Institutional Review Board. All methods were performed in accordance with the relevant guidelines and regulations. IIAM and NORS obtain informed consent from next-of-kin or the donor.

Airway epithelial cells were isolated using a method previously described^30,31^. Briefly, the large airways were dissected out and protease (Sigma Aldrich, St. Louis, MO) digested. After 36–48 hours, the airway lumens were scraped and the resulting cells were plated on collagen-coated tissue culture plates in Bronchial Epithelial Growth Medium (BEGM) (Lonza, Basel, Switzerland).

### Site directed mutagenesis and generation of lentiviral constructs

Recombinant lentiviral plasmids were constructed using the pRRL156.FOXJ1.MCS vector^32,33^ a gift from Dr. Matthias Salathe and Dr. Nevis Friegen at the University of Miami Miller School of Medicine. For the initial construct, a gene encoding PP1α fused to GFP (parent plasmid; pEGFP(C1)-PP1alpha; Plasmid #44224: Addgene, Cambridge, MA) was cloned into the multiple cloning site with XbaI and Mlu1 at the 5′ and 3′ end, respectively, downstream of a *foxj1* promoter to generate pRRL156.FOXJ1.EGFP-PP1α (PP1^WT^). Cysteine 155 to alanine PP1α mutants (PP1^C155A^) were generated using overlap extension PCR with the following forward and reverse primers, respectively: *TTCACTGACGCCTTCAACTGCC* and *GGCAGTTGAAGGCGTCAGTGAAG*, to generate pRRL156.FOXJ1.EGFP-PP1α.C155A (PP1^C155A^). These constructs were confirmed by sequencing. Lentiviruses from these constructs were prepared by the University of Iowa Gene Transfer Vector Core (https://medicine.uiowa.edu/vectorcore/).

### Lentiviral transduction of cultured human airway epithelial cells

Undifferentiated human airway epithelial cells were infected with PP1^WT^ and PP1^C155A^ lentiviruses based on previously described methods^34^. In brief, undifferentiated airway epithelial cells (2^nd^ passage) were seeded onto collagen-coated T-col filters (0.4 μm; Corning, Corning, NY) at 2 × 10^5^ cells per cm^2^ in BEGM containing either virus at MOI = 4 (5 × 10^5^/TU) and 2 μg/ml polybrene (final concentration) and incubated overnight in 37 °C in 5% CO_2_. The following day, virus was discarded and medium was changed to StemCell (Vancouver, Canada) Pneumacult ALI medium in the apical and basal compartments and medium changed every 2 days. When confluent, the apical medium was removed and the cells were maintained in culture until significantly ciliated (3–4 weeks).

### Isolation and experimental treatment of axonemes from hBEC ALI

Immediately following ethanol treatment, axonemes were isolated from cultured hBEC ALI using modifications (see Supplementary Methods) of previously established methods^35^. Axonemes were then diluted to approximately 100–120 motile points per field after addition of activation reagents and then frozen for storage at −80 °C until needed. For motility assays, ATP and activation buffers were added as above with or without the substitution of one part cAMP (10 μM final concentration in resuspension buffer) substituted for resuspension buffer.

### Biotin Switch Method for Detection of S-nitrosation

Protein nitrosation was determined by a modification of the biotin switch as described by Jaffrey *et al*. using the Cayman Chemical S-nitrosated Protein Detection kit (Cayman Chemical; Ann Arbor, MI) to label and detect SNO bonds as per manufacturer’s instructions^16^. To control for background non-specific biotinylation, ascorbate was a not added to a subset of the samples. Biotinylated and input samples were then detected by Western blot as previously described with antibodies for PP1α (anti-mouse; 7482; Santa Cruz Biotechnology; Dallas, TX)^9^, or imaged by immunofluorescence microscopy with co-staining of acetylated α-tubulin (anti-rabbit; 6–11-B1; Santa Cruz Biotechnology; Dallas, TX).

### Ciliary Beat Frequency analysis

CBF was determined using Sisson Ammons Video Analysis (SAVA) as previously described^36^.

### Phosphatase activity assays

Phosphatase activity was determined as previously described using the Ser/Thr Phosphatase Assay Kit 1 (KR-pT-IRR; EMD Millipore; Billerica, MA)^8^. Purified recombinant PP1 (New England Biolabs, Ipswich, MA), isolated axonemes from bovine tracheae or human airway epithelial cells, or tracheal ring lysates were prepared according to the manufacturer’s protocol and treated as indicated prior to adding the K-R-pT-I-R-R peptide. Once the peptide was added the reaction was incubated for 10 min at room temperature and stopped with Malachite Green.

### Protein kinase activity assays

Kinase activity measurement was performed in a manner as previously described^10^. Primary hBEC ALI were prepared by removing apical and basal medium after experimental conditions, adding 250 μl cell lysis buffer and then flash freezing. Samples were thawed and scraped into centrifuge tubes and kept on ice. The cell containing supernatant was sonicated and centrifuged at 10,000 g at 4 °C for 30 min. PKA activity was then measured from the extracted cell or tissue sample. For analysis of isolated bovine or human axoneme PKA activity, axonemes were harvested immediately following experimental conditions and protein content equilibrated (0.3 mg / mL) with axoneme resuspension buffer. Kinase activity was measured in the presence or absence of 10 μM cAMP by a modification of the methods previously described^8^ using a reaction mix consisting of 130 mM PKA heptapeptide substrate in a buffer containing 20 mM Tris–HCl (pH 7.5), 100 μM IBMX, 20 mM magnesium-acetate, and 200 μM [γ−^32^P] ATP.

### Statistical Analysis

Each experiment was performed at least three times and data are expressed as mean ± SEM. Significance was determined by ANOVA or Student’s t test. All statistical analyses were performed by GraphPad Prism software (GraphPad Software, San Diego, CA).

## Electronic supplementary material

Supplementary video 1

Supplementary video 2

Supplementary info
