# Supplementary material for: S-nitrosation of protein phosphatase 1 mediates alcohol-induced ciliary dysfunction
Source: Sci Rep. 2018 Jun 26;8:9701. doi: 10.1038/s41598-018-27924-x (PMC6018795; doi:10.1038/s41598-018-27924-x)

S-nitrosation of protein phosphatase 1 mediates alcohol-induced ciliary dysfunction.

Michael E. Price[1, 2], Adam J. Case[2], Jacqueline A. Pavlik[1], Jane M. Devasure[1], Todd A. Wyatt[1, 3, 4], Matthew C. Zimmerman[2], Joseph H. Sisson[1]

[1]From the Department of Internal Medicine, Pulmonary, Critical Care, Sleep & Allergy Division, [2]Department of Cellular and Integrative Physiology, [3]Department of Environmental, Agricultural, and Occupational Health, University of Nebraska Medical Center, Omaha, NE, USA, [4]Nebraska-Western Iowa VA Healthcare System, Research Service, Omaha, NE, USA

To whom correspondence may be addressed: Joseph H. Sisson, M.D. University of Nebraska Medical Center, Omaha, NE 68198-5300, Tel: (402) 559-5910, Fax: (402) 559-8210, e-mail: jsisson@unmc.edu

Supplementary Information

Methods

Materials and reagents

Recombinant protein phosphatase 1 (rPP1) and Protein Phosphatase Inhibitor 2 (I-2)was purchased from (New England Biolabs; Ipswich, MA). S-nitrosoglutathione was purchased from (ENZO Life Sciences; Farmingdale, NY). 200 proof ethanol was purchase from Decon Laboratories (King of Prussia, PA). $CaCl_2$, Triton X-100, NaCl, $HgCl_2$, DTT, KCl, $MgCl_2$-6H_2O, Sucrose, ATP, Adenosine 3',5'-cyclic monophosphate sodium salt monohydrate (cAMP), KCH_3COO, $MgSO_4$, EDTA, sodium ascorbate, Soybean Trypsin Inhibitor, Tween-20, procaterol hydrochloride, glutathione (reduced), and BioRad TC-20 counting slides were purchased from Sigma Aldrich (St. Louis, MO). BSA, Tris-HCl, Gluthathione (oxidized; GSSG), and neocuproine (1, 10-phenanthroline) were purchased from Thermo Fisher (Waltham, MA).

Isolation and experimental treatment of axonemes from hBEC ALI. Axonemes were isolated from cultured hBEC ALI using modifications as described below using previously established methods [34]. In brief, well-differentiated cultures were first rinsed at least 2 times with cold phosphate buffered saline (PBS). Then cold cilia extraction buffer (20 mM Tris-HCl, 50 mM NaCl, 10 mM $CaCl_2$, 1 mM EDTA, 7 mM β-mercaptoethanol, 0.1% Triton X-100 and 1.0 mM dithiothreitol (DTT)) was added at 64 μl per cm^2 to the apical surface and placed on a plate shaker on high setting for 1 minute. The apical fluid was collected from the cells and placed in a separate tube on ice. This procedure was repeated until there were no more motile points on the culture dish. The extraction buffer containing the axonemes was then centrifuged at 300 x g at 4 °C for 15

s to remove cells and debris. The supernatant was placed in a new tube on ice and centrifuged at 12,000 x g for 5 minutes. The supernatant was discarded and the pellet resuspended in 50 µl resuspension buffer (20 mM Tris-HCl, 50 mM KCl, 4 mM $MgCl_2$-$6H_2O$, 0.5 mM EDTA, 10 mM Soybean Trypsin Inhibitor, 1.0 mM DTT and 25% sucrose). Axonemes were then diluted 6-fold by adding 1 part 15.0 mM ATP (stock concentration), 1 part cilia activation buffer (24 mM Tris-HCl, 60 mM KCH_3COO, 7.2 mM $MgSO_4$, 0.6 mM EDTA and 1.2 mM DTT) and 3 parts resuspension buffer and placed in a TC20 cell counter slide and analyzed by high speed video microscopy. Axonemes were then diluted to approximately 100-120 motile points per field after dilution with activation reagents. Once axoneme motile points were equalized for all samples, ATP and activation buffers were added (see Supplementary methods for detail) with or without the substitution of one part cAMP (10 µM final concentration in resuspension buffer) substituted resuspension buffer.

Western blotting. SDS-PAGE was based on the method previously described using precast 4-20% Tris-HCl gels (Bio-Rad Laboratories; Hercules, CA) [9]. After SDS-PAGE, proteins were transferred to nitrocellulose, which were blocked wash buffer (0.005 M Tris, 0.15 M NaCl, 0.005% Tween-20, pH 7.5) with 3% BSA than incubated with the primary antibodies: PP1α (anti-mouse; Santa Cruz: 7482) in wash buffer overnight at 4°C. The membranes were then washed three times with wash buffer followed by incubation with horseradish peroxidase (HRP) conjugated secondary antibodies (Rabbit anti-Mouse; Sigma (St. Louis, MO) diluted (1:10,000) in wash buffer with 3% BSA for 1 h at room temperature with shaking. Proteins were identified using SuperSignal West femto Substrate (Pierce; Rockford, IL) and exposing the membranes to autoradiography film or a Li-Cor C-Digit Blot Scanner (Lincoln, NE).

Ciliary Beat Frequency analysis. Prior to treatment, samples were removed from the incubator, allowed to cool for 10 minutes on a temperature controlled stage (25 °C), and an initial baseline CBF reading was taken. After treatment the cells were removed from the incubator, allowed to cool for 10 minutes on a temperature controlled stage (25 °C), and a second (baseline) CBF reading was taken. The cells were then treated immediately with 10 nM procaterol by adding a concentrated stock of procaterol to the basal surface and then adding 25 µl basal medium to the apical surface and returned to the incubator. After 50 minutes, the cells were removed from the incubator, allowed to cool for 10 minutes on a temperature controlled stage (25 °C), and a final (stimulated) CBF reading was taken.

Immunofluorescence microscopy of fixed samples. IHC

IHC was based on a previously published method [36]. Briefly, slides were washed twice with PBS and air-dried. After performing the biotin switch technique, ALI membranes were fixed to glass microscopy slides. The cells were then treated with a blocking-permeabilization solution (0.005 M Tris-HCl 0.02% sodium azide, 0.5% Triton X-100 and 1% BSA) for 30 min. The slides were then kept in a humidity chamber for the remaining steps. Cells were incubated with primary antibodies diluted in blocking-permeabilization solution for 1 hr, washed 10 times with TBS + 0.05% Tween-20 (TBS-T), incubated with secondary antibodies diluted in blocking-permeabilization for 1 hr, washed five times with 0.005 M Tris-HCl + 0.01 M NaCl (TBS), and then washed five times with TBS-T. Cells were then mounted in 50 µl Prolong Gold with DAPI (Molecular Probes (Eugene, OR) and coverslips were sealed along the edges with clear nail varnish (Thermo Fisher, Waltham, MA).

Fluorescence microscopy

Fluorescent images were obtained using a Zeiss or Zeiss LSM 510 confocal laser-scanning microscope (Göettingen, Germany) utilizing an argon/krypton laser (488 nm/568 nm/647 nm). Images were collected as .tif files. Fluorochromes used were Alexa 488 and 540.

Supplementary Figures

Movie S1. **Ciliary motility with expression of PP1eGFP.**

Cilia expressing PP1eGFP are visualized at 63X.

Movie S2. **Isolated axoneme motility with expression of PP1eGFP.**

Axonemes were isolated from human airway epithelial cell cultures expressing PP1eGFP. The ciliary membranes were removed with 0.1% Triton X-100, reactivated with ATP and visualized to have motility at 488 nm wavelength and 63X magnification.

Original full-length blot images – Figure 3B

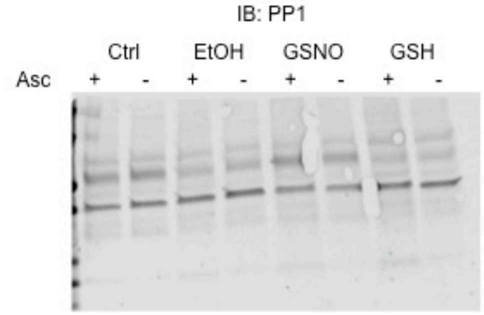

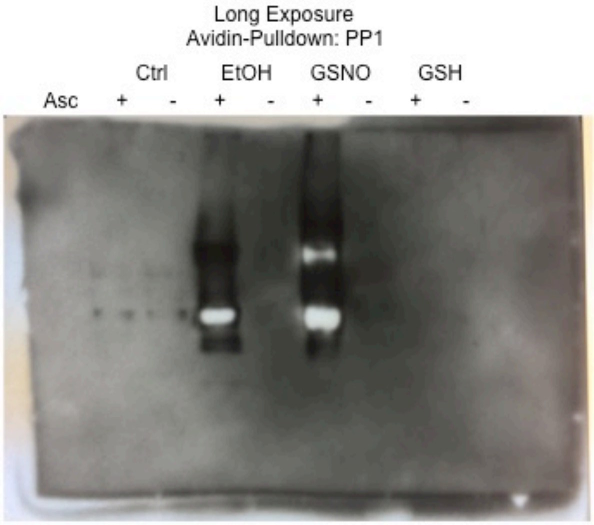

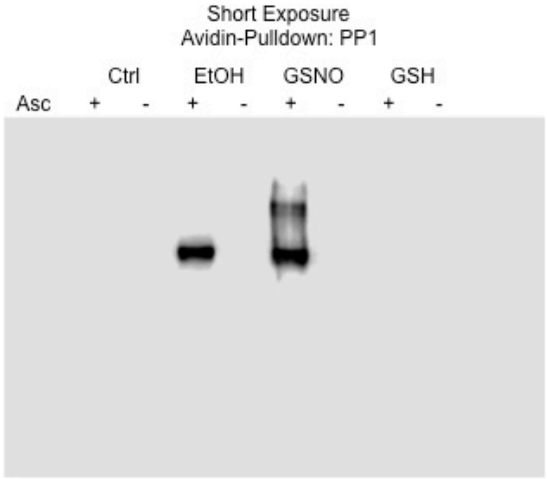

Original full-length blot images – Figure 4C

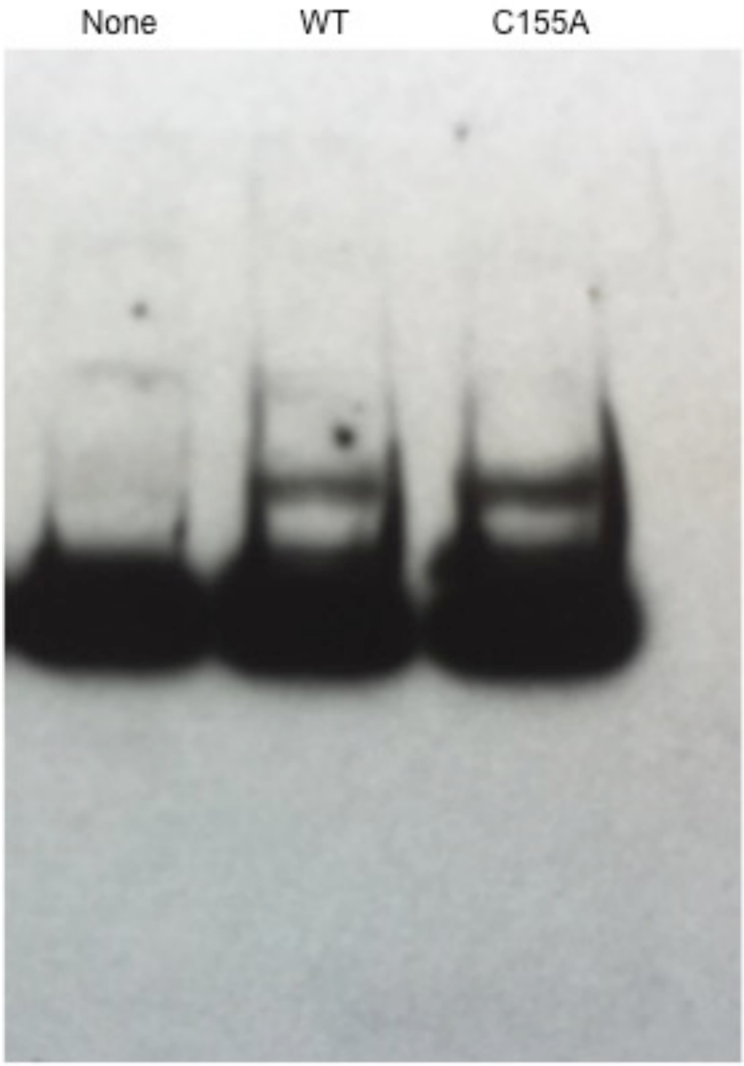

Original full-length blot images – Figure 5B

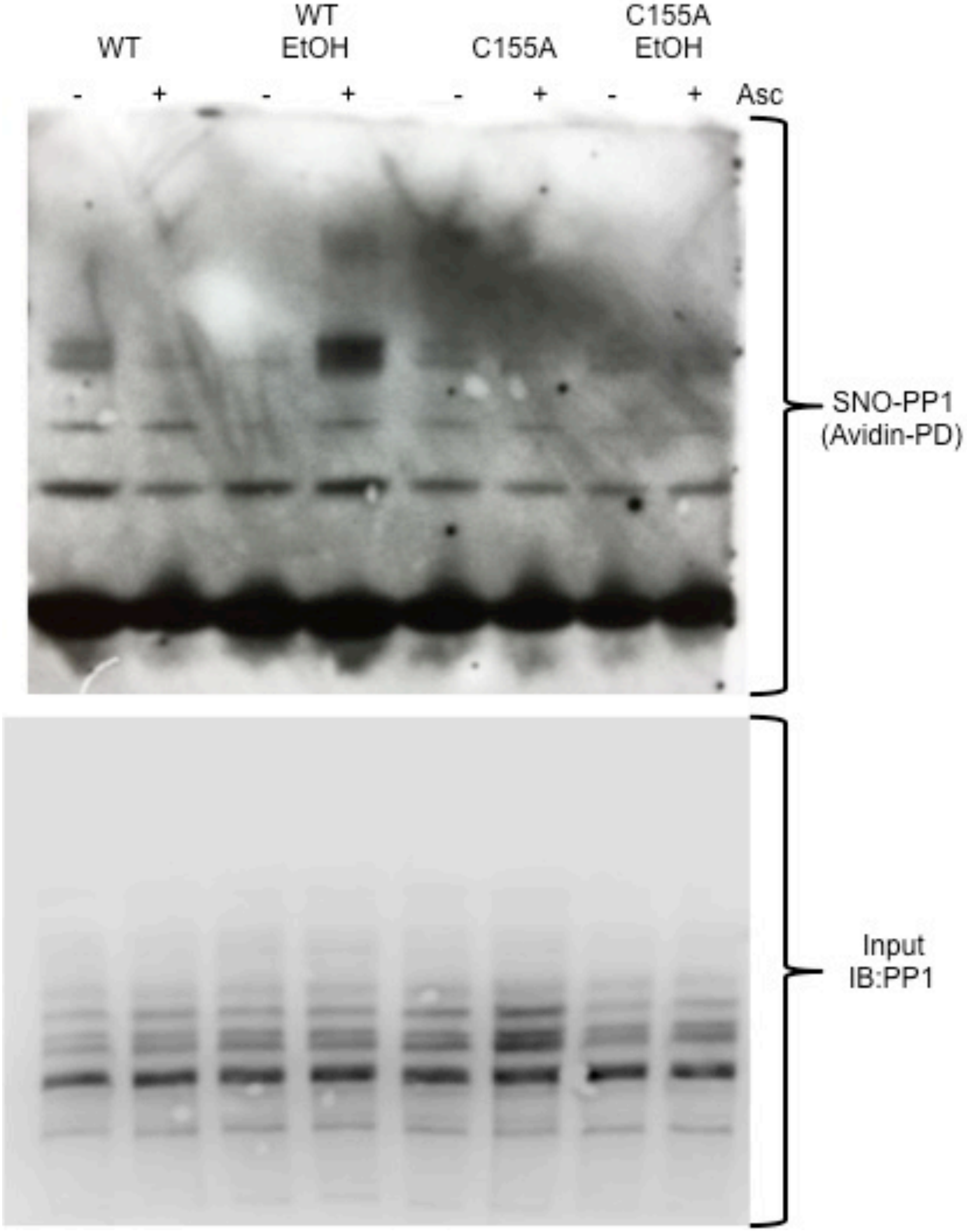

Supplement: Supplementary file 3 — Supplementary info [file 41598_2018_27924_MOESM3_ESM.pdf]
